# Cardiac tamponade due to pneumopericardium 

**Published:** 2014

**Authors:** Yuansheng Xu, Zhijun Xu, Yi Wang

**Affiliations:** 1 Yuansheng Xu, MD, Department of Emergency, Hangzhou First People’s Hospital, Nanjing Medical University Affiliated Hangzhou Hospital, 261 Huansha Road, Hangzhou City, Zhejiang Province, 310006, China.; 2 Zhijun Xu, MD, Department of Emergency, Hangzhou First People’s Hospital, Nanjing Medical University Affiliated Hangzhou Hospital, 261 Huansha Road, Hangzhou City, Zhejiang Province, 310006, China.; 3 Yi Wang, MD, Department of Emergency, Hangzhou First People’s Hospital, Nanjing Medical University Affiliated Hangzhou Hospital, 261 Huansha Road, Hangzhou City, Zhejiang Province, 310006, China.

**Keywords:** Pneumopericardium, Cardiac tamponade, Barotrauma, Portable ventilator

## Abstract

Tension pneumopericardium is an uncommon complication of mechanical ventilaton. It may indeed be life-threatening for hemodynamic compromise and circulatory collapse. We present a case of tension pneumopericardium in a patient with a portable ventilator during intrahospital transport for computed tomography scan. Although timely rescue measures were performed, the patient died finally. We report the case to help us to be aware of and take precautions against this fatal condition during intrahospital patient transportation.

## INTRODUCTION

Pneumopericardium was first described in 1844, and up to 37% of pneumopericardium progress to tension pneumopericardium. Tension pneumopericardium is a rare entity but a life-threatening complication due to subsequent cardiovascular compromise and circulatory collapse. Most of pneumopericardium cases were infants with mechanical ventilation, while its incidence in adult patients is comparatively very little..^1^^,^^2^ We present an adult case developing into tension pneumopericardium during intrahospital transport with portable ventilator. Similar cases have rarely been reported in the earlier literature.

## CASE REPORT

**Fig.1:**
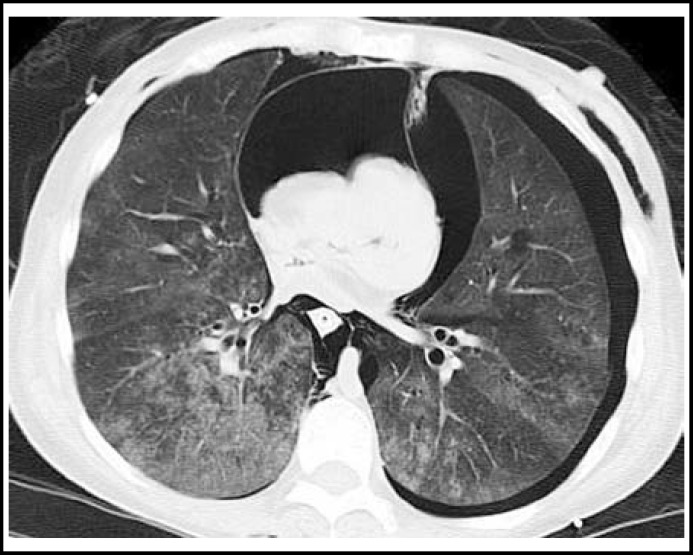
CT showing subcutaneous emphysema, bilateral pneumothorax, tension pneumopericardium and compressed heart

A 60-year-old female patient was admitted with a seven day history of pyrexial, productive cough and increasing breathlessness. On arrival in the emergency room, vital signs were unstable. Arterial blood gas analysis revealed type I respiratory failure. Chest radiograph showed bilateral diffuse patchy opacity and pulmonary consolidation at the right lower lung bases. The diagnosis of acute respiratory distress syndrome (ARDS) was made and endotracheal intubation was performed, ventilation commenced with volume control mode, with tidal volume(VT) 6 ml/kg predicted body weight, respiratory rate(RR) 30 breaths/min, inspiratory time 0.4s, fraction of inspired oxygen (FiO_2_) 0.60, and positive end-expiratory pressure(PEEP) 10 cmH_2_O. Improved oxygenation was gradually maintained. On day 7 the patient underwent a chest computed tomography (CT) scan with a transport ventilator (Osiris 2 made by Air Liquide), which was set as volume assistance or control mode with the upper alarm limitation of peak inspiratory pressure (PIP) 50 cmH_2_O,VT 400ml, RR 20bpm. During the CT examination, the patient`s pulse oxygen saturation decreased below 85% and heart rate decreased gradually. After the termination of scanning, subcutaneous emphysema was found in upper limb, neck and chest. The CT revealed air leak syndrome including massive bilateral pneumothorax, pneumopericardium, pneumomediastinum, pneumoretroperitoneum and subcutaneous emphysema (Fig.1). A puncture needle was immediately inserted into left chest and the air escaped. Percutaneous pericardiocentesis was also performed by the cardiac surgeon later but cardic arrest still occurred. Intravenous epinephrin and cardiopulmonary resuscitation were carried out. Then the patient gradually recovered autonomous cardiac rhythm. However, the patient still remained hemodynamically unstable under intravenous dopamine and norepinephrine. Cerebral resuscitation was unsuccessful due to hypoxic ischemic encephalopathy in spite of improved oxygenation. In the next few hours blood pressure and heart rate dropped progressively without response to vasoactive drugs. The patient eventually deceased for circulatory collapse. Autopsy was not permitted.

## DISCUSSION

Pneumopericardium is defined as the existence of air in the pericardial cavity, it usually arises from trauma or iatrogenic lesion such as barotraumas, endoscopy, and surgical operation. It was not clear how air leaked into the pericardial space and tension pneumopericardium finally occurred. We attributed it to volutrauma or barotraumas induced by mechanical ventilation, in the absence of any other clear explanation because there was no invasive operation procedure before the CT scan, several possible mechanism may contribute to it.

Firstly, severe pnuemonia might result in underlying pulmonary parenchymal pathologic changes and tissue destruction apt to rupture. Pneumonia accompanied with pneumopericardium caused by anaerobic bacteria, Staphylococci and Klebsiella have been described before.^3^ Moreover, mechanical ventilation predisposed alveoli to both barotrauma and volutrauma, which aggravated overdistention of the friable alveoli. Crucially, during intrahospital transport, patient-ventilator dyssynchrony generated a sudden increased excessive peak airway pressure, which ruptured ill alveolus or the terminal bronchioles and then the air dissected the blood vessels, travelled along the peri bronchial or perivascular sheaths and spreaded further to the pericardium, subcutaneous tissues, rarely peritoneum and retro peritoneum. Eventually lethal tension pneumopericardium occurred by one-way valve mechanism and worsened by mechanical ventilation.^4^ When patient-ventilator dyssynchrony, actual peak airway pressure vary dramatically and may overshoot the upper alarm limitation of peak inspiratory pressure because of feedback mechanism delay of air feed cessation and the opening of exhalation valve. Frank GE et al.^5^ have confirmed that sometimes transport ventilator could produce an oscillatory flow during inspiration that lead to rapid changes of the peak airway pressure, which is even more than 50 cmH_2_O in some critically ill patients. It has also been proven that high PIP was associated with an incidence of barotrauma.

The clinical manifestations of the tension pneumopericardium presents with chest distress, chest pain, short breath, and syncope. Physical examination reveals tachycardia and hypotensive, neck vein distention, diminished heart sounds and pulsus paradoxus. The auscultation may find a “mill-wheel” murmur arising from the heart beating in a pericardial cavity filled with air and fluid.^6^

The radiographic findings of pneumopericardium may present “the small heart sign”, “the flattened heart sign”, “the halo sign” outlined by air surrounding the compressed heart.^7^^,^^8^ The CT scan is considerably sensitive to disclose the presence of little indiscernible gas by chest X-ray. Electrocardiography frequently reveals low voltages and other non-specific changes.^9^

It is imperative to identify early tension pneumopericardium and start appropriate treatment. The treatment options also vary in accordance to the severity of the pneumopericardium, ranging from conservative observation or aspiration in mild cases without cardiac tamponade to urgent surgical treatment in severe situations.

It was an uncommon case that pneumopericardium was accidentlly recorded by CT scan. Cardic shock and anoxia attack owing to tension pneumopericardium could be responsible for unsuccessful resuscitation, so a high index of suspicion of air leak syndrome is required during intrahospital transportation with portable ventilator, especially any oxygen saturation drop. Once diagnosis is made, appropriate treatment should be administrated immediately.
